# Understanding the association between injecting and sexual risk behaviors of injecting drug users in Manipur and Nagaland, India

**DOI:** 10.1186/1477-7517-9-40

**Published:** 2012-12-18

**Authors:** Khrieketou Suohu, Chumben Humtsoe, Niranjan Saggurti, Shrutika Sabarwal, Bidhubhusan Mahapatra, Michelle Kermode

**Affiliations:** 1 Emmanuel Hospital Association, Project ORCHID, CIHSR 4th Mile, B Block, 2nd Floor, Dimapur, Nagaland, 797112, India; 2 Population Council, New Delhi, India; 3 Nossal Institute of Global Health, University Melbourne, Victoria, Australia

**Keywords:** Injecting drug users, Sexual behavior, Condom use, Northeastern India

## Abstract

**Background:**

In India, as in rest of the world, HIV prevention programs have focused on HIV transmission through unsafe injecting practices with less attention on sexual risk behaviors among injecting drug users (IDUs). This study examines the sexual risk taking behaviors of IDUs associated with their pattern of drug use in India.

**Methods:**

Data were obtained from the behavioral tracking survey conducted in 2009 among 1712 IDUs in two districts each of Manipur and Nagaland states in Northeastern part of India. Sexual risk behaviors among IDUs were assessed in terms of multiple sex partners, sex with a paid female partner in the last 12 months and inconsistent condom use with any female partner.

**Results:**

More than one-fourth (27%) in Manipur and almost one in two (47%) IDUs reported having had sex with two or more female partners in the past 12 months. In Manipur where heroin is commonly used, the odds of having multiple sex partners were higher among non-heroin users than heroin users (42% vs. 23%, Adjusted Odds Ratio (AOR): 1.7, 95% Confidence Interval (CI): 1.1-2.6) and who shared needles/syringes in the last one month than who did not share (46% vs. 26%, AOR: 2.2, CI: 1.2-4.0). In Nagaland, where Spasmoproxyvon (SP, a synthetic opioid analgesic that contains dextropropoxyphene, dicyclomine hydrochloride and paracetamol) is most common, regular injectors as compared to occasional injectors were more likely to report multiple sex partners (67% vs. 42%, AOR: 2.7, CI: 1.8-4.1) and sex with paid partners (13% vs. 3%, AOR: 6.0, CI: 3.0-12.1). Sharing of needles/syringes was positively associated with multiple sex partners (51% vs. 44%, AOR: 1.6, CI: 1.2-2.2), and inconsistent condom use (93% vs. 80%, AOR: 3.0, CI: 1.8-5.1).

**Conclusions:**

IDUs with unsafe injecting practices also engage in risky sexual practices magnifying the risk of HIV infection. There is a need to focus on prevention of sexual transmission among high-risk IDUs, particularly in areas where Spasmoproxyvon is commonly used.

## Background

Approximately 10% of HIV infections worldwide are attributed to unsafe injecting of illicit drugs [1]. HIV infection, hepatitis C and hepatitis B, all infections linked with injecting drug use, are important public health concerns in India [2-7] and in many other countries worldwide [1,8-10]. The National AIDS Control Organization (NACO) in India has also identified injecting drug users (IDUs) as one of the high risk groups requiring special focus [11].

HIV epidemics among IDUs in the Northeast Indian states of Manipur and Nagaland have been a concern since the mid-1990s. Both states share porous borders with Myanmar, which is part of the Golden Triangle (Myanmar, Laos and Thailand), and is the second largest producer of opium in the world [12]. Approximately 2% of the population of Manipur and Nagaland inject drugs [13]. In Manipur, heroin is the most commonly injected drug whereas in Nagaland Spasmoproxyvon (SP, a synthetic opioid analgesic that contains dextropropoxyphene, dicyclomine hydrochloride and paracetamol) is used more widely. Heroin use became popular among the youth in 1980’s, particularly in Manipur, and unsafe injecting of illicit drugs has remained a major route of HIV infection in Northeast India ever since. The states of Manipur and Nagaland consistently report among the highest HIV prevalence in the country, and in the case of Manipur, the highest. Adult HIV prevalence in 2009 was estimated to be 1.4% in Manipur and 0.78% in Nagaland (compared with 0.31% nationally) [14]. However, HIV prevalence among IDUs is much higher. A large cross-sectional behavioral and biological survey undertaken in two districts within each state in 2009 revealed that HIV prevalence among IDUs ranged between 16.2% - 39.9% in Manipur, and 1.0% - 2.1% in the Nagaland districts.

Avahan (Bill & Melinda Gates Foundation’s HIV initiative in India) has been complementing the HIV prevention programs of the State AIDS Control Societies in these two states through Project ORCHID (Organized Response for Comprehensive HIV Interventions in selected high-prevalence Districts of Manipur and Nagaland), a joint initiative of the Emmanuel Hospital Association (EHA) and the Australian International Health Institute (AIHI) from the University of Melbourne since 2004 [15].

Most of the interventions in India that aim to reduce HIV infection among IDUs have largely focused on transmission of infection through unsafe injecting drug use [14-16]. However, there is also a need to understand and address sexual transmission of HIV infection among IDUs and their sexual partners. While a few studies from Indonesia and United States of America (USA) have demonstrated no association between injecting and sexual risk behaviors [17,18], other international studies conducted in South Africa and USA have established this association [19-23]. Although some Indian studies have investigated the sexual risk behaviors of IDUs [2,4,6,24,25], none have attempted to analyze the associations between injecting and sexual risk behaviors of IDUs in Northeast India, where injecting drugs remains an important route of HIV transmission [13,15]. Understanding this association will assist program planners and managers to sharpen the focus of their HIV prevention interventions. Therefore, this study examines the relationship between injecting and sexual risk behaviors of IDUs in the Indian states of Manipur and Nagaland. It also examines how this relationship is influenced by the type and pattern of drugs injected.

## Methods

### Study design

Data for this study were obtained from a Behavioral Tracking Survey (BTS) conducted in 2009 among IDUs in Ukhrul and Chandel districts of Manipur, and Kiphire and Zunheboto districts of Nagaland. The BTS is a cross-sectional survey that collects information on HIV risk behaviors, HIV knowledge, exposure to Project ORCHID’s harm reduction interventions, and community mobilization. Ethics approval was obtained from the Institutional Review Board of the Emmanuel Hospital Association Ethics Committee, New Delhi.

### Sampling

The Behavioral Tracking Survey used respondent driven sampling (RDS) to recruit participants. RDS is a validated probability sampling method used with hidden populations such as sex workers and IDUs [26]. RDS is based on conventional snowball sampling and is used to recruit participants in many HIV biological and behavioral surveillance studies [27]. RDS involves recruiting participants via an initial pool of accessible contacts (seeds) who are the starting point for a system of recruitment using coupons and financial incentives. A sample size of 400 was estimated based on the ability to detect changes in proportions of 15% at follow-up surveys from estimated baseline values of 50% (which yield the biggest sample size), an alpha level of 0.05 for a two-sided test, and a power of 90%. A design effect of 1.5 was applied to account for intra-class correlation [24]. A total of 1712 participants, 421 from Ukhrul, 415 from Chandel, 427 from Kiphire and 449 from Zunheboto, were recruited for this study.

### Data collection

The eligibility criteria were being male, 18 years of age or older and having injected drugs for non-medical reasons at least once in the last 6 months. Peer educators who had good knowledge of the local injecting drug use context screened the participants for eligibility. Eligible participants were interviewed using a questionnaire that collected information on socio-demographic characteristics, drug use and injection practices, sexual behavior and condom use, knowledge of HIV and exposure to interventions. The questionnaire was adapted from one used previously for a large scale survey in India [28], and was piloted prior to formal data collection.

To start the RDS sampling chain, four seeds in each district were purposively selected to ensure diversity of demographic characteristics including age, geographic location, marital status, employment status, and drug use pattern. An individual coupon system was used to track the recruitment of participants. Seed participants were provided with three coupons each to distribute to fellow IDUs in their social network. Only people presenting the coupon were eligible to participate in the study. Each subsequent participant was also given three coupons and could recruit up to three other IDUs to participate in the study. A primary payment of INR 100 (approx.USD 2.00; 1 USD = INR 50) was provided to each participant at the conclusion of their interview and a secondary payment of INR 50 (approx. USD 1) was provided for each additional participant that they recruited through their three coupons. This process was continued until the desired sample size was attained.

### Measures

The main outcome measures for this study were sex with multiple partners in the last 12 months, sex with a paid partner in last 12 months, and inconsistent condom use with any female partner. The number of sexual partners in the last 12 months was dichotomized into less than two partners and two or more partners. Participants were classified as inconsistent condom users if they reported not always using a condom with any of their female sexual partners (regular, paid, or non-paid casual).

The independent measures considered for this study were frequency and type of drug use, duration since injecting drugs, sharing needles/syringes in the last one month, and accessing services from nongovernmental organizations (NGOs) that had a needle and syringe exchange program (NSEP) in the last six months. Frequency of drug use was dichotomized with regular injectors defined as those IDUs who injected drugs at least daily, whereas occasional injectors were those who injected once or more per week, but not on a daily basis. The type of drug use was different for the two states. The majority of the participants from Manipur had most recently been injecting heroin, whereas the majority from Nagaland had most recently been injecting Spasmoproxyvon. Consequently, for the drug use variable, Manipur participants were coded as heroin users or non-heroin users, and Nagaland participants as Spasmoproxyvon users or non-users. Duration of drug use was dichotomized as less than three years since injecting drugs and three or more years. Sharing of needle/syringe was defined as passing on or receiving a used needle/syringe to or from another IDU.

Socio-demographic characteristics included age, literacy, marital status and employment status. Age was recorded as a continuous variable and categorized into three categories; less than 24 years, 25 to 29 years and 30 years or older. Literacy was assessed as whether the participant could read and write or not. Marital status was categorized as the participant being currently married or not currently married, and employment status was recorded as unemployed if the participant was a student or did not have any source of income.

### Statistical analysis

RDS data are usually analyzed with RDSAT software that generates appropriately weighted estimated proportions with confidence intervals [26,29]. The weights are designed to account for patterns of recruitment. However, RDSAT software is not able to calculate bivariate or multivariate statistics, so we used STATA (Version 11.0) for bivariate and adjusted logistic regression analyses to estimate the association between injecting and sexual risk behaviors of IDUs. Logistic regression models were adjusted for age, literacy, marital status, employment status, and having received needles/syringes from NSEP. These adjusted odds ratios were derived from unweighted estimates. Separate models were developed for each state because of the major differences in the nature of the HIV epidemic, the profile of drug users, and the patterns of drug use in the two states.

## Results

### Socio-demographic characteristics, injecting practices, program exposure and sexual risk behaviors

In both Manipur and Nagaland, the majority of IDUs were literate, 90% and 73% respectively, and were not currently married, 65% and 69% respectively. While 61% of IDUs in Manipur were employed, only 42% in Nagaland were. Differences were observed in the injecting practices of IDUs in Manipur and Nagaland. In Manipur, 73% of IDUs were regular (at least daily) injectors and 79% generally injected heroin, whereas in Nagaland, only 18% of IDUs were regular injectors and 87% generally injected Spasmoproxyvon. About 62% of IDUs in Manipur and 54% in Nagaland had been injecting drugs for three or more years. While 93% of IDUs did not share needle/syringes in Manipur, only 62% did not share in Nagaland. Further, 90% of IDUs in Manipur and 70% in Nagaland had accessed NGO services. Only a small percentage of IDUs in both states reported sex with a paid partner in last 12 months, 7% in Manipur and 5% in Nagaland. Inconsistent condom use with any female partner was common both in Manipur and Nagaland, 86% and 85% respectively (Table 1).

**Table 1:** Socio-demographic characteristics, injecting practices, program exposure and sexual risk behaviors among injecting drug users in Northeastern India

	**Manipur**	**Nagaland**
	**(N = 836)**	**(N = 876)**
**Background characteristics**	% (n)	% (n)
***Socio-demographic characteristics***		
**Age**		
<=24	28.7 (240)	48.4 (424)
25-29	25.2 (211)	30.4 (266)
30+	46.1 (385)	21.2 (186)
**Literate**		
No	10.3 (86)	26.6 (233)
Yes	89.7 (750)	73.4 (643)
**Currently married**		
No	64.7 (541)	68.6 (601)
Yes	35.3 (295)	31.4 (275)
**Employed**		
No	39.1 (327)	58.3 (511)
Yes	60.9 (509)	41.7 (365)
***Injecting drug use practices***		
**Frequency of drug use**		
Occasional	27.0 (226)	81.8 (717)
Regular	73.0 (610)	18.2 (159)
**Heroin user**		
No	21.5 (180)	
Yes	78.5 (656)	
**Spasmoproxyvon user**		
No		13.0 (114)
Yes		87.0 (762)
**Duration since injecting drugs**		
< 3 years	37.9 (317)	46.2 (404)
3+ years	62.1 (519)	53.8 (471)
**Shared needle/syringe in last one month**		
No	92.5 (773)	62.0 (543)
Yes	7.5 (63)	38.0 (333)
***Program exposure***		
**Accessed NGO services in last 6 months**		
No	10.2 (85)	25.0 (219)
Yes	89.8 (751)	75.0 (657)
***Sexual risk behavior***		
**Number of female partners in last 12 months**		
<2	72.7 (608)	53.1 (465)
2+	27.3 (228)	46.9 (411)
**Sex with paid partner in last 12 months**		
No	93.5 (782)	95.0 (832)
Yes	6.5 (54)	5.0 (44)
**Inconsistent condom use with any female partners**		
No	13.8 (85)	15.0 (118)
Yes	86.2 (531)	85.0 (670)

### Association between injecting and sexual risk behaviors by state

In Manipur, frequency of drug use and duration of injecting had no significant association with any of the sexual risk behavior indicators. However, heroin users were less likely to have had two or more partners in the last 12 months compared to non-heroin users (23% vs. 42%, Adjusted Odds Ratio (AOR) = 0.6, 95% Confidence Interval (CI) = 0.4-0.9). Further, those who shared needle/syringes in the last one month were more likely to have had two or more partners in the last 12 months compared to those who did not share (46% vs. 26%, AOR = 2.2, 95% CI = 1.2-4.0) (Table 2).

**Table 2:** Associations between pattern and type of injecting drug use, exposure to program and sexual risk behaviors among injecting drug users in Northeastern India

**Characteristics**	**2+ partner in last 12 months**		**Sex with paid partner in last 12 months**		**Inconsistent condom with any female partner**	
	**%**	**AOR**^**1 **^**(95% CI)**	**%**	**AOR**^**1 **^**(95% CI)**	**%**	**AOR**^**1 **^**(95% CI)**
**Manipur (N = 836)**						
**Frequency of drug use**						
Occasional	26.1 (226)	Referent	6.6 (226)	Referent	85.5 (173)	Referent
Regular	27.7 (610)	0.9 (0.6-1.4)	6.4 (610)	0.8 (0.4-1.6)	86.5 (443)	1.2 (0.7-2.1)
**Heroin user**						
No	41.7 (180)	Referent	3.3 (180)	Referent	84.5 (142)	Referent
Yes	23.3 (656)	**0.6 (0.4-0.9)**	7.3 (656)	2.2 (0.9-5.6)	86.7 (474)	1.0 (0.5-1.8)
**Duration since injecting drugs**						
< 3 years	31.2 (317)	Referent	8.5 (317)	Referent	81.0 (221)	Referent
3+ years	24.9 (519)	1.1 (0.8-1.6)	5.2 (519)	0.6 (0.3-1.0)	89.1 (395)	1.4 (0.9-2.4)
**Shared needle/syringe in last one month**						
No	25.7 (773)	Referent	6.3 (773)	Referent	85.7 (561)	Referent
Yes	46.0 (63)	**2.2 (1.2-4.0)**	7.9 (63)	1.7 (0.6-4.6)	90.9 (55)	1.8 (0.7-4.9)
**Accessed NGO services in last 6 month**						
No	20.0 (85)	Referent	3.5 (85)	Referent	92.8 (69)	Referent
Yes	28.1 (751)	1.5 (0.7-3.1)	6.8 (751)	1.2 (0.2-5.4)	85.4 (547)	1.0 (0.3-3.7)
**Nagaland (N = 876)**						
**Frequency of drug use**						
Occasional	42.4 (717)	Referent	3.2 (717)	Referent	85.8 (633)	Referent
Regular	67.3 (159)	**2.7 (1.8-4.1)**	13.2 (159)	**6.0 (3.0-12.1)**	81.9 (155)	0.8 (0.5-1.3)
**Spasmoproxyvon user**						
No	45.6 (114)	Referent	5.5 (762)	Referent	84.9 (680)	Referent
Yes	47.1 (762)	0.9 (0.6-1.4)	1.8 (114)	3.4 (0.8-14.8)	86.1 (108)	1.3 (0.7-2.5)
**Duration since injecting drugs**						
< 3 years	46.5 (404)	Referent	5.2 (404)	Referent	77.4 (337)	Referent
3+ years	47.3 (471)	1.4 (0.9-1.9)	4.9 (471)	1.2 (0.6-2.4)	90.7 (450)	**1.7 (1.1-2.9)**
**Shared needle/syringe in last one month**						
No	44.2 (543)	Referent	5.0 (543)	Referent	80.1 (477)	Referent
Yes	51.4 (333)	**1.6 (1.2-2.2)**	5.1 (333)	0.9 (0.5-1.7)	92.6 (311)	**3.0 (1.8-5.1)**
**Accessed NGO services in last 6 months**						
No	31.5 (219)	Referent	5.0 (219)	Referent	95.3 (193)	Referent
Yes	52.1 (657)	**2.7 (1.7-4.2)**	5.0 (657)	1.0 (0.2-2.5)	81.7 (595)	**0.4 (0.2-0.9)**

95% CI- 95% Confidence Interval.

AOR-Adjusted Odds Ratio.

^1^Adjusted for age, literacy, marital status, employment status and received needle/syringe from an NSEP.

In Nagaland, regular injectors were more likely to have had two or more partners in the last 12 months (67% vs. 42%, AOR = 2.7, 95% CI = 1.8-4.1) and more likely to have had sex with a paid partner in the last 12 months (13% vs. 3%, AOR = 6.0, 95% CI = 3.0-12.1). Those IDUs who had been injecting for three or more years were more likely to report overall inconsistent condom use compared to those who had been injecting for less than three years (91% vs. 77%, AOR = 1.7, 95% CI = 1.1-2.9). The IDUs who reported sharing needle/syringes in the last month reported having two or more partners in the last 12 months (51% vs. 44%, AOR = 1.6, 95% CI = 1.2-2.2) and overall inconsistent condom use (93% vs. 80%, AOR = 3.0, 95% CI = 1.8-5.1) as compared to those who did not share in the last month. Although a higher proportion of IDUs who accessed services reported having two or more partners in the last 12 months as compared to those who did not access services (51% vs. 32%, AOR = 2.7, 95% CI = 1.7-4.2), a smaller proportion reported inconsistent condom use (82% vs. 95%, AOR = 0.4, 95% CI = 0.2-0.9).

## Discussion

The results indicate that a significant proportion of injecting drug users in both the states of Manipur and Nagaland engage in high risk sexual behaviors. Further, IDUs who share needles were more likely to have multiple sexual partners. However, state level differences in the association between injecting and sexual risk behaviors were also evident, and the observed differences could be, at least in part, due to the different types of drugs used in each of the states. In Manipur, heroin users were less likely to have two or more partners in the last 12 months as compared to non heroin users. Further, sex with a paid partner in the last 12 months was not associated with any injecting behavior of IDUs. This is consistent with literature that documents a negative association between the use of heroin and sexual risk behavior [18] as heroin use tends to suppress sexual desire [23,30]. In Nagaland, Spasmoproxyvon was used more widely and our results demonstrate that riskier injecting behavior was associated with riskier sexual behavior in this state. Although regular injectors in Nagaland were more likely to have two or more partners in the last 12 months and have sex with paid partners, those who accessed NGO services were significantly less likely to be inconsistent condom users. Hence NGO services have been effective in increasing condom use among those IDUs who actually access them, as documented by a previous study conducted in these two states [24]. These findings persisted even after adjusting for socio-demographic characteristics known to be strong confounders of both injecting and sexual behaviors. Our finding that IDUs practicing high risk injecting behaviors are more likely to practice high risk sexual behaviors is consistent with findings from previous studies conducted in the USA and South Africa [19-21,23].

The present study findings should be considered in light of the following limitation. As the data for each district was collected independently and an unweighted analysis conducted at the state level by combining districts, we could not use RDSAT for our regression analysis. Further, since there is no consensus among statisticians as to whether data gathered through RDS can be appropriately weighted for multivariate analysis, it is important to interpret these regression findings with some caution [31].

Despite this limitation, the findings highlight the double jeopardy faced by high risk IDUs who engage in both risky injecting and risky sexual practices, amplifying not only their own risks of HIV infection but also the likelihood of HIV transmission to their injecting and sexual partners. This underscores the importance of interventions that focus on safer sexual practices, especially among high risk IDUs, in the ongoing HIV prevention programs in Northeast India.

This is especially the case in Nagaland where the link between unsafe injecting and unsafe sex was strongest. The importance of preventing sexual transmission of HIV in Nagaland is being increasingly recognized. In 2008, the HIV prevalence among IDUs in Nagaland was only 3.2% (compared with 9.2% among IDUs nationally), while among FSWs it was 14.1% (compared with 4.9% among FSWs nationally) [32]. Based on prevention of parent to child transmission (PPTCT) testing data, Nagaland has the highest HIV prevalence among pregnant women in the country (0.89% compared with 0.19% nationally) [32]. These data point towards a shift away from an HIV epidemic driven by unsafe injecting, to one driven by unsafe sexual behaviors. The overlap between unsafe injecting and unsafe sexual behaviors among a high-risk sub-set of IDUs, as identified in this study, has the potential to amplify HIV transmission in this state.

Typically, programs targeted towards IDUs focus primarily on distribution of needles and syringes; however, as documented in previous literature [33], it is essential that these efforts are strongly complemented with sexual risk reduction programs. Also, given that condom use is more consistent among IDUs who access NGO services, extending the coverage of the targeted HIV prevention interventions so they reach a greater proportion of IDUs is recommended.

## Abbreviations

AIHI: Australian international health institute; AOR: Adjusted odds ratio; BTS: Behavioral tracking survey; CI: Confidence interval; EHA: Emmanuel hospital association; HIV: Human immunodeficiency virus; IDU: Injecting drug user; NACO: National AIDS Control Organization; NGO: Nongovernmental organization; NSEP: Needle and syringe exchange program; ORCHID: Organized response for comprehensive HIV interventions in selected high-prevalence districts of Manipur and Nagaland; PPTCT: Prevention of parent to child transmission; RDS: Respondent driven sampling; USA: United States of America.

## Competing interests

The authors declare that they have no competing interests.

## Authors’ contribution

KS was responsible for conceptualization and writing of the paper; CH led research implementation and assisted in interpretation of study findings; NS assisted in conceptualization and interpretation of study findings; SS conducted literature review and assisted in drafting of manuscript; BM performed the statistical analysis and assisted in writing of manuscript; MC provided overall guidance. All authors have read and approved this version of the manuscript.
